# Biophysical insights from a single chain camelid antibody directed against the Disrupted-in-Schizophrenia 1 protein

**DOI:** 10.1371/journal.pone.0191162

**Published:** 2018-01-11

**Authors:** Antony S. K. Yerabham, Andreas Müller-Schiffmann, Tamar Ziehm, Andreas Stadler, Sabrina Köber, Xela Indurkhya, Rita Marreiros, Svenja V. Trossbach, Nicholas J. Bradshaw, Ingrid Prikulis, Dieter Willbold, Oliver H. Weiergräber, Carsten Korth

**Affiliations:** 1 Department of Neuropathology, Heinrich Heine University Düsseldorf, Düsseldorf, Germany; 2 Institute of Complex Systems (ICS-6: Structural Biochemistry), Forschungszentrum Jülich, Jülich, Germany; 3 Jülich Centre for Neutron Science JCNS and Institute for Complex Systems ICS, Forschungszentrum Jülich, Jülich, Germany; 4 Institute for Physical Biology and BMFZ, Heinrich Heine University Düsseldorf, Düsseldorf, Germany; Scuola Internazionale Superiore di Studi Avanzati, ITALY

## Abstract

Accumulating evidence suggests an important role for the Disrupted-in-Schizophrenia 1 (DISC1) protein in neurodevelopment and chronic mental illness. In particular, the C-terminal 300 amino acids of DISC1 have been found to mediate important protein-protein interactions and to harbor functionally important phosphorylation sites and disease-associated polymorphisms. However, long disordered regions and oligomer-forming subdomains have so far impeded structural analysis. V_H_H domains derived from camelid heavy chain only antibodies are minimal antigen binding modules with appreciable solubility and stability, which makes them well suited for the stabilizing proteins prior to structural investigation. Here, we report on the generation of a V_H_H domain derived from an immunized *Lama glama*, displaying high affinity for the human DISC1 C region (aa 691–836), and its characterization by surface plasmon resonance, size exclusion chromatography and immunological techniques. The V_H_H-DISC1 (C region) complex was also used for structural investigation by small angle X-ray scattering analysis. In combination with molecular modeling, these data support predictions regarding the three-dimensional fold of this DISC1 segment as well as its steric arrangement in complex with our V_H_H antibody.

## Introduction

*Disrupted- in-schizophrenia 1* (*DISC1*) was discovered as the gene disrupted by a balanced chromosomal translocation (1; 11) (q42.1; q14.3), which is strongly linked with schizophrenia and several other mental disorders in a large Scottish family [1]. In an American family with mental illness, a frame shift mutation following amino acid 807 in the DISC1 protein was reported [2]. DISC1 is known to interact with more than 150 other proteins [3], but has no known enzymatic activity and is considered to act as a scaffolding protein, regulating the functions of its binding partners [4]. DISC1 is involved in cerebral cortex development and affects critical processes including neuronal migration and proliferation [5, 6]. Studies on animal models have associated pathological DISC1 protein variants with behavioral abnormalities [7], which were linked to disturbances in various neurotransmitter systems including dopamine [8, 9].

The C-terminal region comprising amino acids 598–854 of the human DISC1 protein (UniProtKB Q9NRI5) contains interaction sites for several binding partners, such as nuclear distribution element 1 (NDE1), NDE-like 1 (NDEL1), and lissencephaly 1 (LIS1, encoded by the *PAFAH1B1* gene), which are important for brain development and neuronal migration [10, 11]. It also harbors functionally important sites such as the mental illness associated S704C polymorphism [12–14] and a phosphorylation site at S713, which was found to coordinate the switch of neuronal progenitor cell proliferation to migration during corticogenesis [15]. Moreover, a DISC1 protein fragment consisting of amino acids 598–785 was shown to possess unique characteristics such as cell invasiveness [16, 17] and to contribute prominently to the structural order of the DISC1 protein, while amino acids 668–747 are essential for an orderly assembly of oligomers [18]. Recent findings regarding the structural organization of the DISC1 protein showed that the C-terminal portion of the DISC1 protein covers three out of the four known structural regions of the protein: the I region (aa 539–655), the S region (aa 635–738) and the C region (aa 691–836) [19]. The I region was shown to be the most aggregation-prone of these regions, whereas the S region is very stable and highly oligomeric. The C region, which was previously thought to be dimeric, was proved to exist as a monomer but to lose its high solubility due to the disease related frameshift mutation at amino acid 807 with nine novel amino acids. Therefore, structural information on the C-terminal portion of the DISC1 protein is desirable for understanding the role of this region in the protein’s normal function, solubility, and interaction properties, as well as the effects of truncations found in disease variants.

Single-domain camelid antibodies, also called V_H_H domains or nanobodies, are engineered polypeptides used in a large range of applications [20]. They consist entirely of the variable domain (V_H_) of heavy chain-only immunoglobulins, which occur in the sera of animals belonging to the Camelidae family. This variable domain alone is completely functional in recognizing a specific antigen, without the presence of a paired light chain variable (V_L_) domain [21–23].

The numerous benefits of V_H_H fragments compared to conventional antibodies include higher yields of expression when produced as recombinant proteins in bacterial cells, higher levels of stability and solubility, and greater ease of delivery for therapeutic applications, while retaining high affinity and specificity towards the target antigen [24]. V_H_H antibodies have proven applicable in diverse fields, from therapeutic interventions and diagnostics to basic research, including the investigation of protein structure and function [25–28].

Here we report the generation of the first V_H_H antibody directed against human DISC1, termed B5. Using biochemical and biophysical methodology, we quantitatively characterize the interaction of the antibody with DISC1 and determine its epitope. Moreover, the structures of the DISC1 C region [19] and its complex with V_H_H B5 are investigated by small angle X-ray scattering (SAXS) analysis and molecular modeling. These results provide insight into the binding mode of our V_H_H antibody to the DISC1 protein.

## Experimental procedures

### Llama immunization

A female llama was immunized on days 0, 17, 32, and 65 with 240 μg of purified recombinant human DISC1 fragment comprising residues 598–785 (DISC1 598–785) [18] in 0.5 mL PBS, mixed with an equal volume of complete (day 0) or incomplete (all other days) Freund’s adjuvant. At day 70, 550 mL of blood was taken and used to generate a phage library. The immunization procedure and blood withdrawal from the llama was contracted to the company Preclinics GmbH (Potsdam, Germany). All animal research was authorized by the Niedersächsische Landesamt für Verbraucherschutz und Lebensmittelsicherheit (LAVES), Oldenburg, Germany (Az 33.9-42502-05-09A618) and conducted according to the European Directive for Animal Research.

### Construction of the phage library

500 mL of blood was collected 7 days after the final injection and diluted 1:1 with PBS. In total 6.9 × 10^8^ lymphocytes were isolated via Ficoll Paque gradient centrifugation, pelleted and snap frozen in liquid nitrogen before storage at -80°C.

RNA was extracted using the RNeasy Mini Kit (Qiagen, Germany) and first strand cDNA was generated with Oligo(dT)18 primers and the Revert Aid M-MuLV Reverse Transkriptase Kit (Thermo Scientific). cDNA encoding V_H_H was specifically amplified by PCR using the sense and anti-sense hinge-specific primers “MJ 1.2.3 Back” and “CH2 + CH2b3”, as described in ref. 29. PCR bands between 620 and 670 bp were eluted from the gel and reamplified by nested PCR using the primers MJ7 [29] and J_H_ [30]. These primers included respective *Sfi*I and *Not*I cleavage sites for subsequent cloning into the phagemid vector pIT2 (obtained from the human single fold scFv Tomlinson I+J libraries, Cambridge, UK) [31]. Ligated DNA was electro-transfected into electro-competent *E*. *coli* TG1 cells. The colonies of plated cells were counted, collected and frozen in growth medium containing 15% glycerol at -80°C. The size of the library was determined to be 1.2 × 10^7^ CFU (colony-forming units).

### Phage display for selection

The phage display process was performed based on the Tomlinson I + J protocol [32]. The phage library generated was used for three rounds of panning in order to screen for the strongest DISC1-binding clone. The panning procedure was performed by coating the Nunc Maxisorp ELISA plates (Thermo Scientific, Denmark) with 0.1 mg/mL of the DISC1 protein fragment aa 598–785 in PBS buffer by overnight incubation at 4°C. The plates were blocked with 1% skimmed milk powder and 1% Casein (Hammerstein grade) in PBS buffer, for alternate rounds of panning, after coating with the DISC1 protein by similar incubation conditions as for coating. The phage library was panned onto these coated and blocked ELISA plates in the respective blocking solution, which were washed 8–10 times with PBS before eluting the final binding phages. Elution was performed using 10 mg/mL trypsin solution dissolved in PBS. The trypsin-eluted phage species from each round of panning were propagated in TG1 *E*. *coli*. The efficacy of each panning round was titrated by colony count at different dilutions of the propagated TG1 *E*. *coli* species infected by the trypsin eluted phages. Typically, the titer of the TG1 *E*. *coli* cells infected with trypsin eluted phages increased in each consecutive round, corresponding to a successful selection process. Thereafter the V_H_H sequence of the clone selected through three rounds of panning from the phage library was cloned into the plasmid vector pET-22b (by *Nco1* and *Not1*) for ease of overexpression.

### Cloning and production of anti-DISC1 human Fc-V_H_H antibody

The anti-DISC1 V_H_H antibody sequence was PCR amplified from the pET-22b vector using forward primer 5’-agacggctgtgtcttcaggt-3’ and reverse primer 5’-acctgaagacacagccgtct-3’. *Age1* and *Not1* sites were added to the PCR product using the forward 5’-gaaaaccggtatggcccaggtaaag-3’ and reverse 5’-cttggcggccgcctactatgcggccgctg-3’ primers. The sequence was then ligated into the expression vector pLHCX-F_C_-fs, downstream of the IgG signal sequence and followed by the human Fc coding sequence.

The expression vector pLHCX-F_C_-fs was generated from a modified pLHCX vector (Clontech), where the multiple cloning site was modified using *HindIII* and *ClaI*, and a synthetic sequence (5’-accggtctcgaggcggccgcggccaaaaaggccggatccgttaacaccaaaaaatggcacgtggccggcacg cgtgggcccgtcgac-3’) was inserted.

To obtain the coding sequence of the human F_C_, mammalian cells were transiently transfected with the pLHCX-PrP-Fc vector, containing the genomic human F_C_ sequence [33]. RNA and derived cDNA were used to clone the coding sequence of the human F_C_ into the modified pLHCX vector in-frame at the *SalI* restriction site. The IgG signal sequence in expression vector pLHCX-F_C_-fs was cloned at the *HindIII* and *BamHI* restriction sites.

The pLHCX-F_C_-fs vector encoding the antibody was used to generate retroviruses according to the manufacturer’s protocol (Clontech), which were used to infect HEK293 cells, leading to stable expression of the V_H_H antibody. HEK293 cells were then cultured in DMEM containing 5% fetal calf serum, penicillin, streptomycin and hygromycin. Antibody secreted into the conditioned media (cell supernatant) was collected and used in subsequent steps.

### Purification of DISC1 protein fragments

A human DISC1 fragment comprising residues 598–785 (DISC1^598-785^) was expressed and purified as described previously [16]. The plasmid vectors pESPRIT002 DISC1 691–836, pESPRIT002 DISC1 539–655, pESPRIT002 DISC1 480–721, pESPRIT002 DISC1 598–715 [19] were used to transform BL21 AI cells. These were then grown in Terrific Broth (12 g tryptone, 24 g yeast extract, 4 ml glycerol autoclaved in 900 mL H_2_O, followed by addition of 100 mL filter sterilized 0.17 M monopotassium phosphate and 0.72 M dipotassium phosphate solution). Protein expression was induced at OD_600_ = 0.8 by the addition of 0.2% L-arabinose and 1 mM IPTG for 16 hours at 25°C. Bacterial pellets were stored at -80°C and lysed by incubation in 25 mM Tris pH 7.4/150 mM NaCl/5 mM imidazole/1 mM DTT/0.5% Triton X-100/20 mM MgCl_2_ containing 0.25 mg/mL Lysozyme, DNaseI 40 U/mL and 2 mM PMSF at room temperature. The insoluble fraction was spun down by centrifugation at 6000 × g for 45 min. The soluble fraction was then incubated with Ni^2+^-NTA-Agarose (Qiagen) for 45 min at room temperature and washed with 25 mM Tris pH 7.4/150 mM NaCl/5 mM imidazole/1 mM DTT. Protein was eluted with the same buffer containing 500 mM imidazole and was then further purified by size exclusion chromatography (SEC) on a HiLoad 16/60 Superdex 200 pg column (GE Healthcare Bio-Sciences AB, Sweden) with a flow rate of 1 mL/min at 4°C.

### Purification of V_H_H B5 antibody

The pET22b V_H_H-B5 plasmid vector was transformed into the BL21 Rosetta strain (Novagen, USA) and protein expression was induced at OD_600_ = 0.8 with 1 mM IPTG at 18°C for 16 hours. Bacterial pellets were stored at -80°C and lysed by incubation in 25 mM Tris pH 7.4/150 mM NaCl/5 mM imidazole/0.5% Trition-C100/20 mM MgCl_2_ containing 0.25 mg/mL Lysozyme, DNaseI 40 U/mL and 2 mM PMSF at room temperature. The insoluble fraction was spun down by centrifugation at 6000 × g for 45 min. The soluble fraction was then incubated with Ni^2+^-NTA-Agarose (QIAGEN) for 45 min at room temperature and washed with 25 mM Tris pH 7.4/150 mM NaCl/5 mM imidazole. Protein was eluted with the same buffer containing 500 mM imidazole and was then further purified by size exclusion chromatography (SEC) on a HiLoad 16/60 Superdex 200 pg column (GE Healthcare Bio-Sciences AB, Sweden) with a flow rate of 1 mL/min at 4°C.

### Monoclonal antibodies and cell lines

The anti-human DISC1 human monoclonal antibody 14F2 has been described previously [17]. The mouse-Disc1 C-term monoclonal antibody was produced in rabbit as described previously [8, 34]. The 6x-His Epitope Tag antibody was purchased from Thermo Scientific.

The NLF human neuroblastoma cell line (Children’s Hospital of Philadelphia) was transfected with human and mouse DISC1. The transfections were performed with 8 μg of plasmid using Metafectene (Biontex; Martinsried, Germany) according to manufacturer’s protocols.

### Surface plasmon resonance (SPR)

SPR measurements were performed using a Biacore T200 instrument (GE Healthcare, Sweden) at 25°C with PBS/0.05% Tween-20, pH 7.4 as running buffer. For preparation of the flow cells, a CM5 sensor chip (GE Healthcare, Sweden) was activated with 1-ethyl-3-(3-dimethylaminopropyl) carbodiimide (EDC) / N-hydroxysuccinimide (NHS) (0.2 M / 0.05 M), the DISC1 fragment comprising residues 691–836 (DISC1^691-836^) (30 μg/mL) diluted in 10 mM sodium acetate, pH 4.0. It was immobilized to a final level of 250 RU, and the flow cell was deactivated with 1 M ethanolamine-HCl. A reference flow cell was activated and deactivated only. Afterwards, V_H_H B5 at concentrations ranging from 19 nM to 1.5 μM was injected in a single cycle without regeneration. The sensorgrams were double referenced using the reference flow cell and a buffer cycle, while evaluation was performed by plotting the respective response levels against the applied V_H_H B5 concentrations. The curves were fitted using Langmuir's 1:1 binding model (Hill function with n = 1, OriginPro 8.5G, OriginLab, Northampton, USA).

### Small angle X-ray scattering data acquisition and processing

SAXS data were recorded using protein samples of >95% purity, as judged by SDS-PAGE with Coomassie staining. In addition to the DISC1^691-836^ protein and the V_H_H B5 antibody, we investigated their complex after co-purification via SEC. SAXS was measured on beam line P12 at the PETRA III storage ring (DESY, Hamburg, Germany [35]) and on BM29 at the ESRF (Grenoble, France [36]). The X-ray wavelengths used on P12 and BM29 were 1.24 Å and 1 Å, respectively. An automatic robot was used for sample storage and loading of the solutions in the quartz capillary for X-ray exposure. Storage and measurement temperature was 20° C throughout all experiments. On P12 the exposure time was 100 ms and 20 frames were recorded, while on BM29 the exposure time was 1 s and 10 frames were taken. Buffer only was measured before and after each protein sample. The individual frames were checked for the absence of radiation damage and the corresponding frames were merged. The scattering contribution of the buffer was subtracted from the measured intensities of the protein solutions. The buffer-subtracted SAXS data were scaled by the protein concentrations, as determined via absorbance at 280 nm. Final datasets were derived from measurements at protein concentrations of 2.8 mg/mL and 2.3 mg/mL for the DISC1^691-836^ protein fragment and the complex with V_H_H B5, respectively, whereas in case of the V_H_H domain B5 recordings for 2.8 and 5.6 mg/mL were merged.

Data were analyzed using the *ATSAS* software package [37]. The radius of gyration (*R*_*g*_) was derived from the Guinier approximation, as implemented in AUTORG [38], while CRYSOL [39] was used for calculation of theoretical values from atomic coordinates. The distance distribution function *p(r)* was determined using the program *DATGNOM* [38]. For each dataset, 20 *ab initio* reconstructions were generated using *DAMMIF* [40] or *GASBOR* [41], followed by averaging and filtering in *DAMAVER* [42]. The resulting consensus models were used in further analyses. For the purposes of fitting and visualization, they were converted into volumetric maps with *pdb2vol*, which is part of the *Situs* package [43].

### Preparation of structural models

A starting model of the DISC1^691-836^ protein fragment, including an N-terminal His tag, was generated by *ab initio* prediction using *QUARK* [44]. This model provided a reasonable fit to the SAXS reconstruction, with a normalized spatial discrepancy (NSD) value of 1.83 as determined by *SUPCOMB [45]*. While it yielded a very good 3D-1D profile, with 92% of all residues scoring higher than the confidence threshold of 0.2 as given by *Verify3D* [46], validation with *QMEAN* [47] indicated problems with torsion angles, corresponding to an overall *QMEAN* score of 0.54 and a Z-score of -2.29. Since visual examination of the model revealed poor geometry in several loop regions, it was subjected to iterative rebuilding using *MODELLER* [48], making extensive use of loop refinement algorithms. This procedure resulted in further improvement of the 3D-1D profile (94% of residues exceeding 0.2) as well as an increase of the *QMEAN* score to 0.67 (Z-score -0.98). The final model comprises all 170 residues of the protein (including residues 691–836 of the human DISC1 sequence). According to validation in *Coot* [49], all residues are located in the allowed regions of the Ramachandran diagram and do not display rotamer outliers. Compared to the initial *QUARK* model, the refined version features a better fit to the SAXS envelope, with an NSD of 1.36.

A homology model for the V_H_H B5 antibody was built in *MODELLER*, using as a template the crystal structure of a llama nanobody (PDB ID 3EZJ, chain B [50]). This model contains residues 1–120, again without Ramachandran or rotamer outliers, and performs well in consistency tests using *Verify3d* (100% scoring > 0.2) and *QMEAN* (score 0.77, Z-score 0.30).

The models of the DISC1^691-836^ protein and the V_H_H B5 antibody were subjected to *in silico* docking simulations using *CLUSPRO* [51] in antibody mode [52]. The resulting models were examined for consistency with the SAXS reconstruction of the complex as well as experimental data narrowing down the binding site, and the best candidate selected for further analysis.

### Fitting to SAXS envelopes

The *ab initio* SAXS reconstructions obtained from *DAMAVER* were initially fit to the corresponding structural models using *SUPCOMB*, which takes care of the handedness ambiguity of the SAXS envelopes. After conversion of the latter to *Situs*-format volumetric maps, the relative positioning of the model structures was optimized by correlation-based refinement, as implemented in *Sculptor* [53].

### Data visualization

SAXS data were plotted using *Grace* (http://plasma-gate.weizmann.ac.il/Grace). Ribbon representations were generated with *POVScript+* [54] and *Raster3D* [55], applying secondary structure assignments provided by *DSSP* [56].

## Results

### Generation of an anti-DISC1 camelid single chain V_H_H antibody

An anti-DISC1 camelid V_H_H phage library was generated using mRNA obtained from a llama that had been immunized with an insoluble fraction of recombinant human DISC1^598-785^ protein. This fragment forms multimers and may be largely responsible for the formation of pathogenic misassembled DISC1 protein [18]. The insoluble DISC1^598-785^ protein had been purified and refolded from inclusion bodies, following overexpression in *E*. *coli*. The phage library generated was then subjected to three rounds of panning by phage display in order to select the strongest binding clone against the DISC1^598-785^ protein. There was a gradual increase in the phage titer in subsequent rounds of panning (3 × 10^3^ CFU/ml, 5 × 10^3^ CFU/ml, 1 × 10^7^ CFU/ml, respectively), indicating a successful selection process.

**Fig 1:**
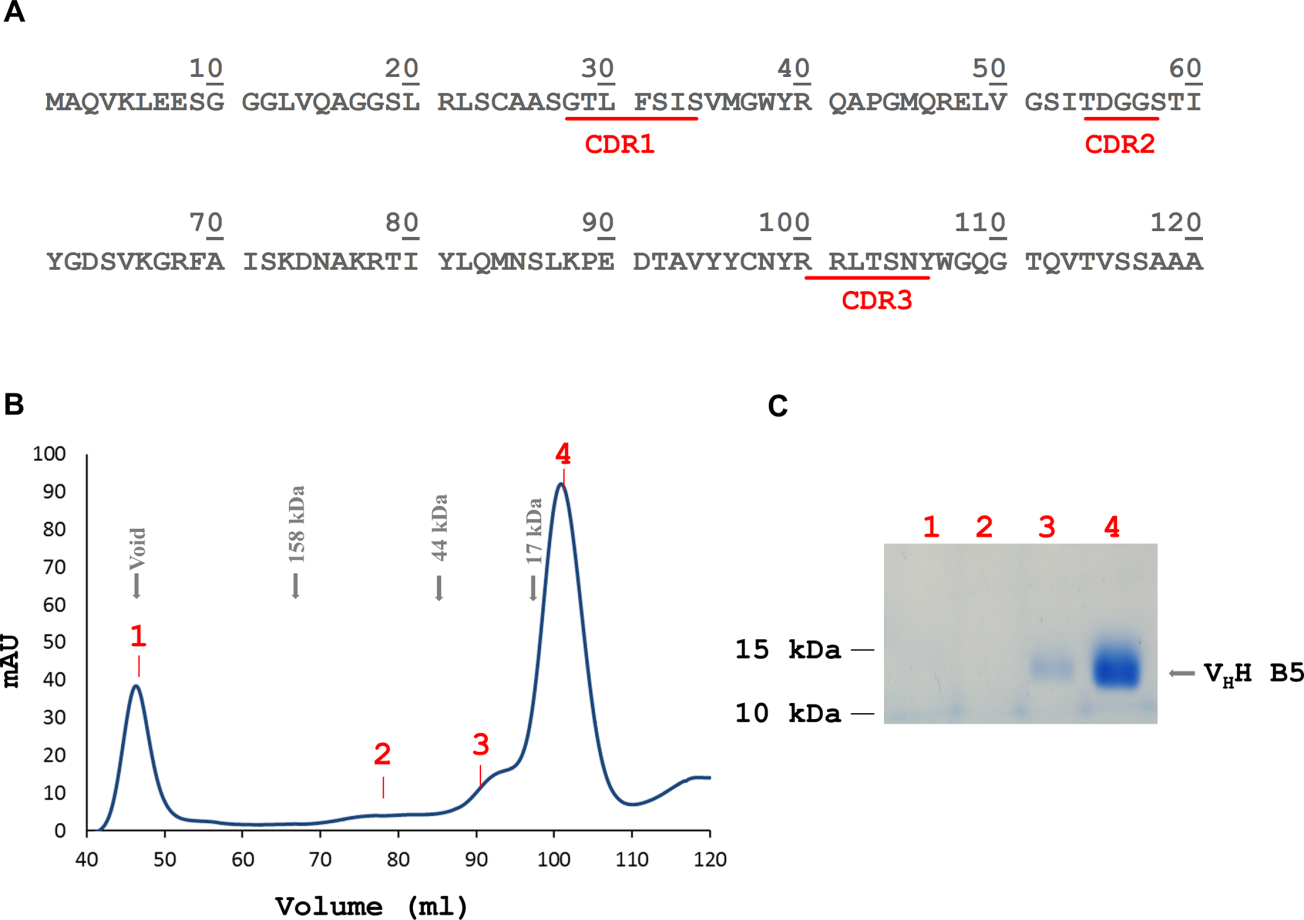
Sequence and purification of the generated anti-DISC1 V_H_H B5 antibody. (A) Sequence of the anti-DISC1 V_H_H B5 protein showing the three complementarity determining regions (CDRs). (B) SEC profile of the purified anti-DISC1 V_H_H B5 antibody eluting with an apparent molecular mass of 13 kDa. (C) Coomassie-stained SDS gel loaded with different SEC elution fractions (samples numbered in accordance with the chromatogram). Fraction 1 corresponds to the void volume, fractions 3 and 4 contain the V_H_H B5 antibody.

The strongest binder obtained, clone B5, was isolated and subcloned into the bacterial expression vector pET-22b (Fig 1A). The camelid V_H_H antibody was then overexpressed as a recombinant protein in the periplasm of *E*. *coli*, providing an oxidizing environment for proper folding, and was then purified by Ni^2+^-NTA affinity chromatography, followed by SEC. In SEC, the V_H_H B5 antibody protein eluted prominently as a monomer and, very minimally, in an oligomeric state (Fig 1B and 1C).

### Interaction of V_H_H B5 with the DISC1^691-836^ protein

In order to confirm and further narrow down the binding of V_H_H B5 to DISC1, we tested for its binding to the C region of DISC1 protein by SEC. DISC1^691-836^ was referred to as ‘C region’ in the structural organisation of the protein proposed recently [19]. This C region is a stable, distinct structural region within the DISC1 protein and is known to exist as a monomer, unlike the immunogen used (DISC1^598-785^), and also holds several physiologically relevant sites such as the disease variant S704C and the crucial phosphorylation site at S713 [15].

**Fig 2:**
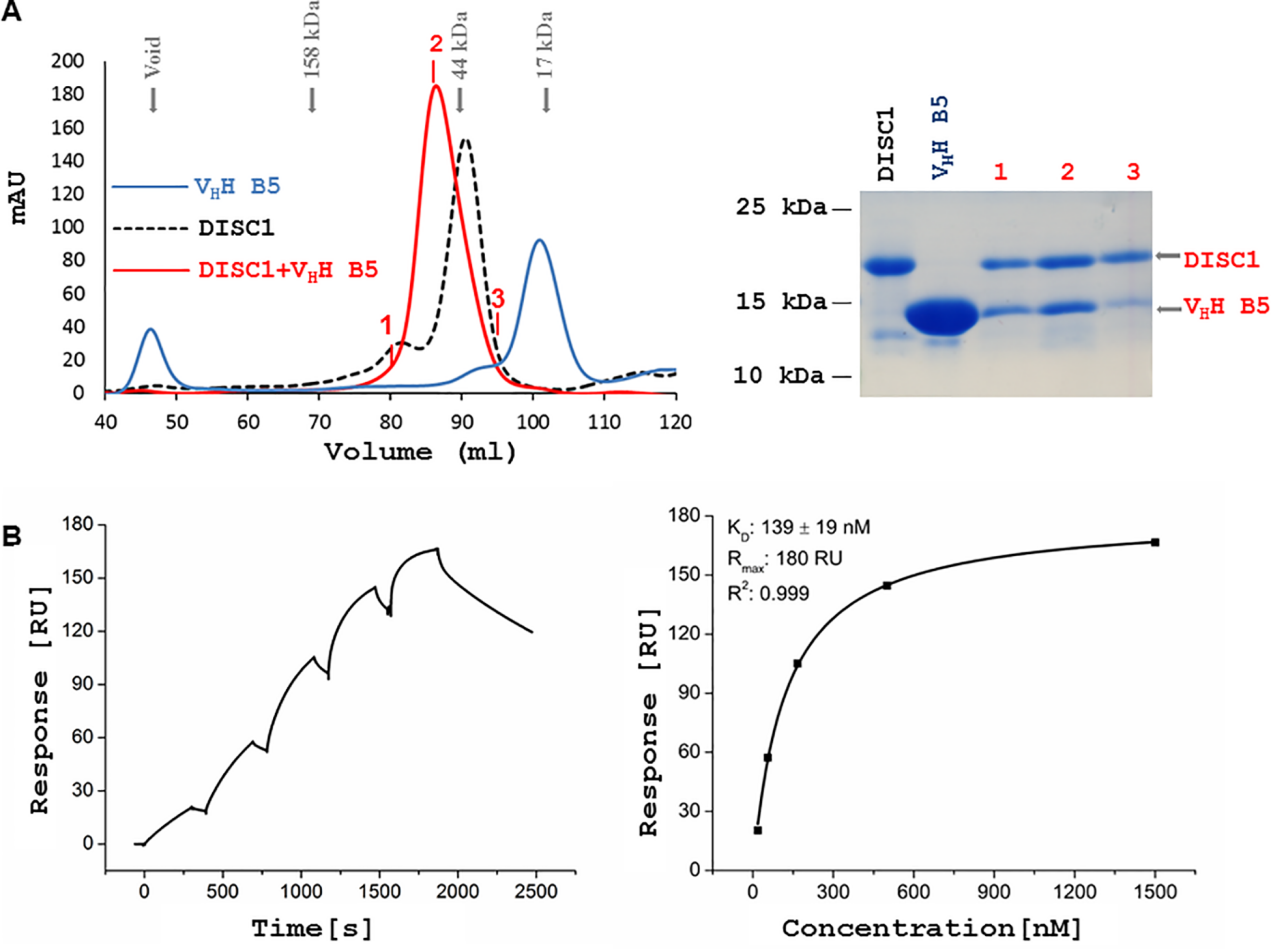
Interaction of the V_H_H B5 antibody with DISC1^691-836^ protein. (A) The SEC profile demonstrates a shift in the elution peak of the DISC1 fragment and V_H_H B5 antibody mixture, compared to the individual proteins. Fractions containing the DISC1-V_H_H B5 co-elution peak (labelled 1, 2 and 3) were investigated by SDS-PAGE. (B) Representative SPR sensorgram showing the binding of a V_H_H B5 antibody concentration series to immobilized DISC1^691-836^ protein and the corresponding fitting curve. The K_D_ value is presented as mean ± SD of three independent experiments.

An equimolar mixture of the DISC1^691-836^ protein and the V_H_H B5 antibody exhibited a shift of the SEC elution peak towards a higher apparent molecular mass when compared to the individual proteins, and the two species were confirmed to co-elute as a complex (Fig 2A), indicating binding of the V_H_H antibody to the DISC1 protein fragment. The binding observed by SEC was further confirmed through SPR, with the affinity of the B5 antibody to the DISC1^691-836^ protein being characterized by a dissociation constant (K_D_) of 139 nM (Fig 2B).

### Mapping of the V_H_H antibody epitope on the DISC1 protein

**Fig 3:**
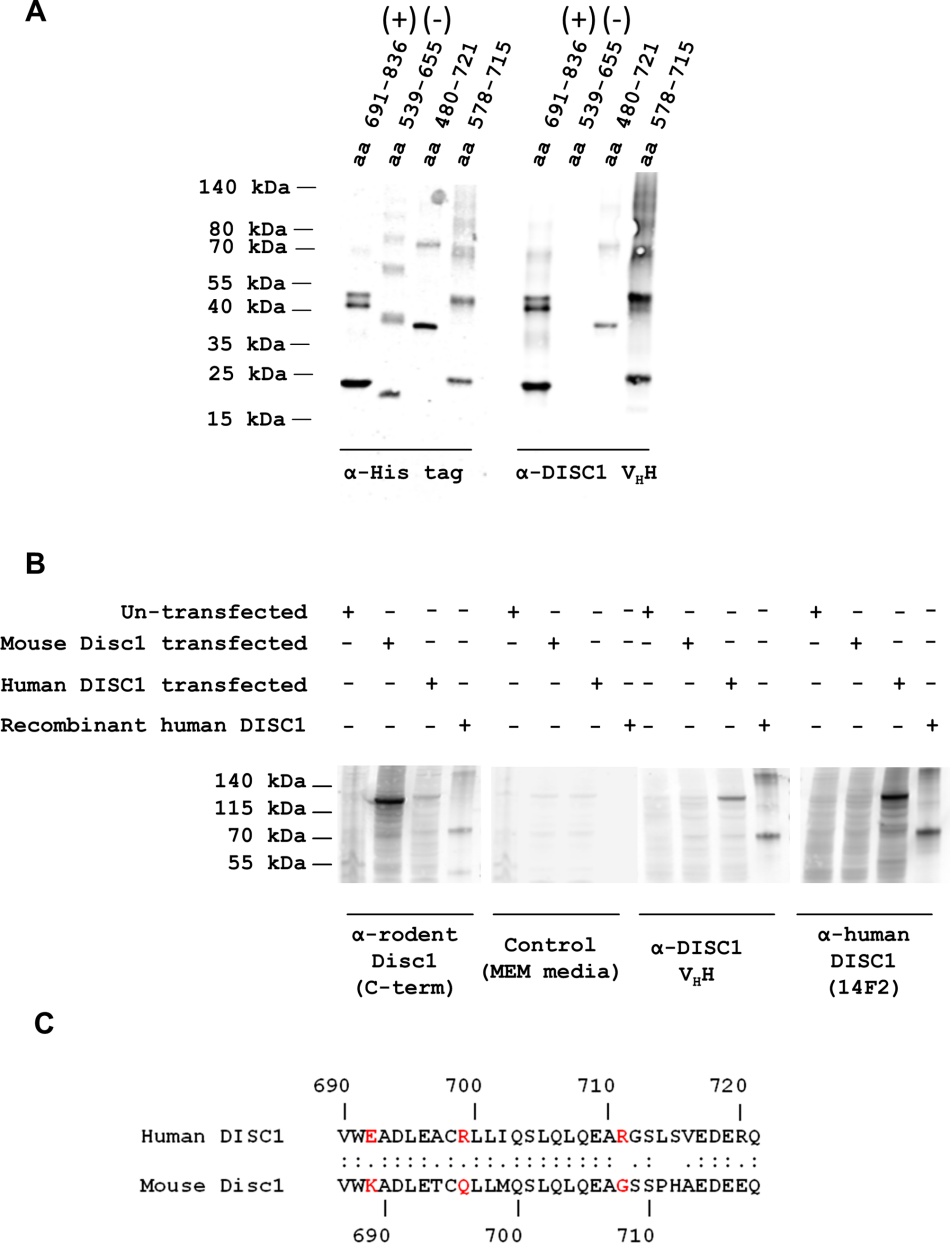
Mapping the binding site of the anti-DISC1 V_H_H B5 antibody on the DISC1 protein. (A) The binding epitope of the anti-DISC1 V_H_H B5 antibody was mapped by western blot using truncated DISC1 protein constructs. (+) and (-) indicate positive and negative control, respectively. All constructs were detected using an anti-His tag antibody, whereas only the constructs covering the 691–715 segment were detected using the anti-DISC1 V_H_H B5 antibody. The multiple bands with higher apparent mass observed in each lane correspond to SDS-resistant oligomers, which are a typical feature of DISC1 protein fragments [19]. (B) Detection of human and mouse DISC1 protein in the lysates of transfected NLF cells by western blot, using either anti-rodent Disc1 C-term antibody, control MEM media, anti-DISC1 V_H_H B5 antibody or anti-human DISC1 14F2 antibody. The four samples tested are untransfected NLF cell lysate, mouse Disc1 transfected NLF cell lysate, human DISC1 transfected NLF cell lysate, recombinant human DISC1 (50 ng). (C) Alignment of the human DISC1 epitope (residues 691–715) with the corresponding region of mouse Disc1.

In order to increase the avidity, as well as to expand the potential applications for functional analysis, the V_H_H B5 was cloned into the plasmid vector pLHCX-F_C_-fs. This allows the secretion, from transfected cells, of a dimeric humanized heavy chain only antibody containing V_H_H B5 as the variable region. We first used this V_H_H–Fc to further narrow down the binding epitope within DISC1, using DISC1 truncation constructs. Several DISC1 truncation constructs obtained previously through ESPRIT (a high-throughput screening technique to identify soluble regions within any protein) [19], DISC1^691-836^, DISC1^539-655^, DISC1^480-721^, and DISC1^598-715^, were probed by western blot using an anti-human secondary antibody. Here DISC1^691-836^ and DISC1^539-655^ were the positive and negative controls respectively, whereas DISC1^480-721^ and DISC1^598-715^ were the test samples. The constructs DISC1^691-836^, DISC1^480-721^ and DISC1^598-715^ were clearly detected by the V_H_H–Fc antibody, indicating the binding site to be contained within the residue range 691–715 (Fig 3A). Similarly, NLF neuroblastoma cells transfected with full length human DISC1 or mouse Disc1 were used to test the specificity of the V_H_H-Fc antibody. Intriguingly, only human DISC1 could be detected and not mouse Disc1 (Fig 3B). An amino acid sequence alignment of the two proteins (UniProtKB Q9NRI5 and Q811T9) in the 691–715 region (Fig 3C) reveals high overall similarity, with non-conservative exchanges (human > mouse) at positions 692 (E > K), 699 (R > Q), and 711 (R > G). The interaction of our V_H_H B5 with human DISC1 is thus likely to involve at least one of these charged residues.

### SAXS analysis of the DISC1^691-836^ protein

**Fig 4:**
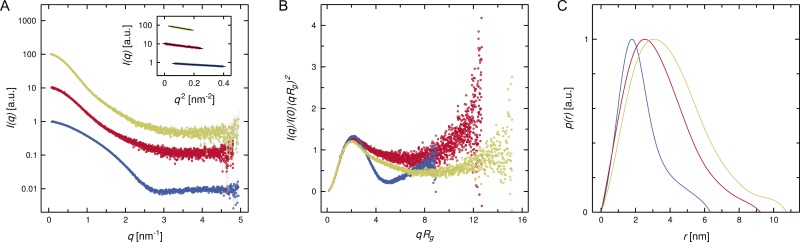
Investigation of DISC1-V_H_H complex formation by SAXS. (A) Primary scattering data (Intensity *I* as a function of momentum transfer *q*, the latter being defined as 4π sinθ / λ) for the V_H_H domain (blue), the DISC1^691-836^ protein (red), and their complex (yellow). The inset shows the linear regions of the respective Guinier plots at low *q* values, as suggested by *AUTORG*; the slope of the linear fit is related to the radius of gyration of the respective sample. (B) Normalized Kratky plot of the data shown in (A). A peak at low *qR*_*g*_ values is considered typical of folded globular proteins. (C) Distance distribution functions derived from the data shown in (A), using the indirect Fourier transformation implemented in *DATGNOM*. For each species, the intersection with the positive abscissa corresponds to the maximum particle diameter. A.u., arbitrary units.

In order to further characterize complex formation between the DISC1^691-836^ protein and the V_H_H B5, SAXS experiments were performed. As shown in Fig 4A, the shapes of the scattering curves differ significantly between the V_H_H B5 on the one hand and the DISC1^691-836^ protein (as well as the complex) on the other. Indeed, the two components are expected to attain very different structures; while immunoglobulin domains are roughly ellipsoidal and mostly contain beta structure, the DISC1^691-836^ protein is predicted to form several alpha helices with different lengths, resulting in a more elongated and possibly irregular shape [19]. In all three cases, the Guinier plot features a linear segment at low momentum transfer (inset), as expected for (essentially) monodisperse samples; the radius of gyration (*R*_*g*_) can be derived from the slope of the respective linear fit, yielding values of 2.64 nm, 1.81 nm, and 3.07 nm for the DISC1^691-836^ protein, the V_H_H antibody and their complex, respectively. Furthermore, the normalized Kratky plots of the primary scattering data (Fig 4B) display a positive peak in the lower *qR*_*g*_ range (ideally at √3, for particles perfectly obeying the Guinier approximation), suggesting a compact three-dimensional fold. However, the subsequent trough is far more pronounced for the V_H_H domain than for DISC1^691-836^, which indicates that the structure of the latter is less ordered. As expected from its composition, the complex features an intermediate behavior. Finally, the different sizes and shapes of the three entities are also obvious from the real-space distance distribution function (Fig 4C) obtained by indirect Fourier transformation. Approximate volumes (and corresponding molecular masses) of the hydrated species can be determined by applying the Porod equation; in accordance with the *R*_*g*_ values, the complex appears to be significantly larger (76.8 nm^3^/45.2 kDa) than either the DISC1^691-836^ protein (40.9 nm^3^/24.1 kDa) or the V_H_H antibody (21.3 nm^3^/12.5 kDa) alone. Hence, our SAXS analysis is consistent with the binding of the V_H_H domain B5 to the DISC1^691-836^ protein in solution. We note, however, that the size of the complex estimated by this method significantly exceeds the sum of the values for the two individual proteins. Given that both SEC and SPR results support a 1:1 stoichiometry, concordant with the expected binding mode of a monoclonal nanobody: this discrepancy is most likely due to a limited degree of oligomerization which was not detectable in the Guinier plot. A more extensive list of quantities determined from the SAXS data is provided in Table 1.

**Table 1:** Parameters derived from the SAXS data (MM, molecular mass).

	DISC^691-836^	V_H_H domain	DISC-V_H_H complex
*R*_*g*_ from Guinier eq. [nm]	2.64	1.81	3.07
*R*_*g*_ from *p(r)* [nm]	2.68	1.85	3.20
*D*_*max*_ from *p(r)* [nm]	9.24	6.34	10.74
*I*_*0*_ from Guinier eq.^a^	17.31	15.15	36.20
*I*_*0*_ from *p(r)*^a^	16.86	13.68	36.35
Porod volume [nm^3^]	40.9	21.3	76.8
MM from Porod vol. [kDa]	24.1	12.5	45.2
MM theoretical [kDa]	19.4	12.9	32.3

^a^ Indicates approximate molecular mass (in kDa) since data is scaled by sample concentration.

## Discussion

In this study, we report the first single domain antibody against the human DISC1 protein. V_H_H B5 was raised against the DISC1^598-785^ fragment. Following recombinant expression and purification, binding of the V_H_H B5 to the DISC1 protein C region (aa 691–836, previously identified to constitute a stable and soluble domain [19]) was demonstrated by three independent methods: size exclusion chromatography, surface plasmon resonance spectroscopy, and small angle X-ray scattering analysis. The binding epitope of our anti-DISC1 V_H_H B5 antibody was determined to reside between residues 691 and 715 of human DISC1. Moreover, the antibody was shown to be specific for human DISC1 and could not detect the mouse Disc1 protein when expressed in transfected NLF cells. The SAXS data on DISC1^691-836^ confirmed our previous findings concerning its monomeric form and extended shape.

**Fig 5:**
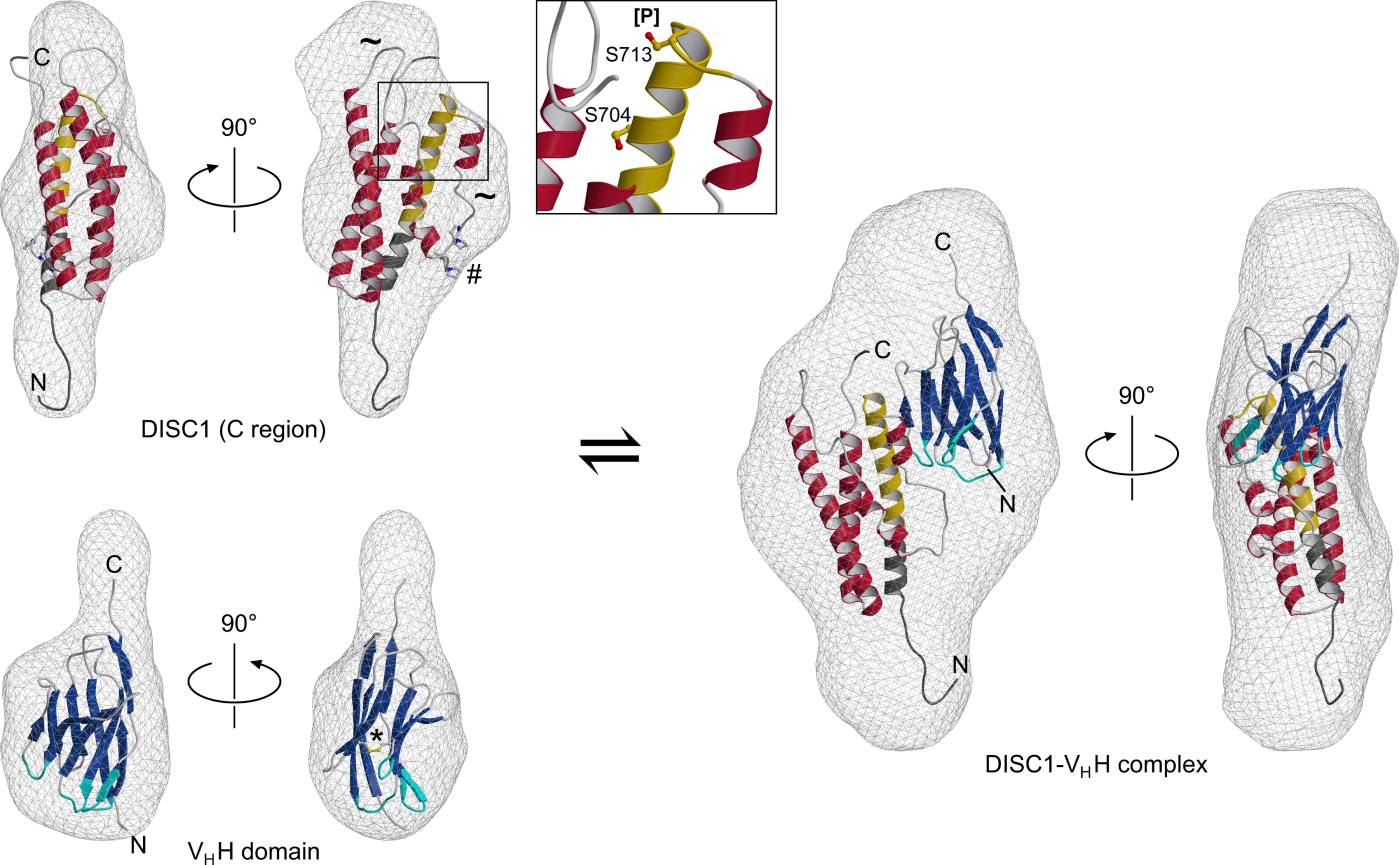
*Ab initio* SAXS reconstructions and molecular models for the DISC1^691-836^ protein, the V_H_H domain, and their complex. The hypothetical DISC1 model (upper left) features a single domain containing three longer and three shorter α-helices. Similar to the terminal segments, the two extended loops (tilde symbols) are suggested to be poorly ordered. The positions of functionally important residues S704 and S713 are indicated in a close-up view. Also note the proline-rich motif (ball-and-stick representation, marked by a hash), which is predicted to be readily accessible for protein-protein interactions. The 691–715 region, suggested to participate in the epitope for the nanobody, is highlighted in gold while cloning artifacts at the termini are colored dark gray. The model of the V_H_H antibody (lower left) displays a canonical immunoglobulin fold, including a conserved disulfide bridge (ball-and-stick model, marked by an asterisk). The CDR segments, as defined by the Chothia criteria [60], are highlighted in cyan. Finally, the DISC1^691-836^ fragment and the nanobody are predicted to interact in a complex (right) featuring a 1:1 stoichiometry.

In addition to quantitative parameters related to the size of the scattering particles, such as *R*_*g*_ and Porod volume, the reciprocal space diffraction profiles or, alternatively, the real-space distance distribution functions (Fig 4) can be used to calculate *ab initio* reconstructions, which essentially indicate the outer shape of the scattering particles. These envelopes are shown in Fig 5 for the DISC1^691-836^ protein, V_H_H B5, and their complex. Moreover, we have developed molecular models to illustrate the structures of these polypeptides and their potential mode of interaction. It is important to note that the Protein Data Bank does not contain any structures with obvious sequence similarity to DISC1, precluding conventional homology modeling. We therefore resorted to the *ab initio* approach implemented in *QUARK* [44] (for details refer to *Experimental Procedures*). While this type of structure assembly is inherently less reliable than template-based approaches, the DISC1^691-836^ protein should represent a relatively favorable case due to its moderate size and predicted abundance of helical secondary structure [57]. Indeed, the resulting model is dominated by a bundle of three long α-helices, the N-terminal one being connected to the other two by a stretch containing three shorter helical segments (Fig 5, upper left). In addition to the termini, two extended loops (residues 723–737 and 752–771) are suggested to display significant conformational freedom. Of these, the 723–737 segment is particularly noteworthy since it contains a proline-rich motif (^730^PPIPP^734^), proposed to function as docking site for the Grb2 SH3 domain, based on mutational analyses [58]. Importantly, the presence of flexible regions in the DISC1^691-836^ fragment is not only consistent with our SAXS investigation outlined above, specifically the shape of the Kratky plot, but is also supported by CD spectroscopy data indicating significant disorder in the 718–771 region [19].

Additional sites with known functional relevance in human DISC1 include S704 and S713, both of which are shown in a close-up view in Fig 5. Position 704 is altered by a single-nucleotide polymorphism leading to an exchange for cysteine, which has been associated with mental illness [12]. According to our model, it is located in the first long α-helix and would not seem to be available for protein-protein interactions. Note that studies investigating the consequences of the S704C mutation revealed only moderate effects on the oligomerization propensity of C-terminal DISC1 fragments [18, 59]; this observation supports the notion that S704 may not be solvent-exposed. S713, on the other hand, has recently been demonstrated to be targeted by at least one kinase *in vivo*, and its phosphorylation is thought to mediate the switch in DISC1 function in neurodevelopment from a proliferation-promoting to a migration-promoting state [15]. This residue is located in the loop adjacent to the first helix and is predicted to be readily accessible. In addition to a change in size and charge of the serine side chain itself, phosphorylation is likely to impart some degree of local rearrangement (possibly translating into long-range effects) due to the presence of basic side chains in its vicinity. It is reasonable to assume that such effects underlie the switch in the DISC1 interactome associated with S713 phosphorylation.

In contrast to the DISC1^691-836^ protein, the structure of the V_H_H B5 domain could be readily modeled based on a template with high sequence similarity (PDB ID 3EZJ). The nanobody displays the canonical V-type immunoglobulin fold, a sandwich composed of a five-stranded and a four-stranded β-sheet, which are connected by a conserved disulfide bridge. As shown in Fig 5, both models fit the SAXS-derived envelopes reasonably well, with NSD values of 1.36 and 1.40 for the DISC1^691-836^ protein and the V_H_H B5 antibody, respectively. We also developed a model of the DISC1-V_H_H B5 complex via *in-silico* docking simulations. The arrangement displayed in Fig 5 is in good agreement with our *ab initio* SAXS envelope, and is also consistent with experimental evidence indicating that core interaction determinants are located within residues 691–715 region of DISC1. Specifically, the CDR loops of the nanobody may contact residue R699 or, with a moderate shift in position, E692; both of these charged residues are exchanged in mouse Disc1, possibly supporting the specificity of our V_H_H antibody for the human orthologue. A comprehensive analysis of the DISC^691-836^-V_H_H interface, however, would be beyond the scope of the *in-silico* efforts reported here, but will have to await experimental determination of the complex structure, for example by X-ray crystallography.

We anticipate that V_H_H B5 will perform favorably in a wide range of potential applications. It should constitute an efficient DISC1 probe for use in *in vitro* and *in vivo* investigations and specifically target the S and C regions, which we have recently suggested to represent structural units of the DISC1 protein [19]. A relevant prospect would be to utilize V_H_H B5 in order to gain mechanistic insight into the aggregation of the DISC1 disease caused by a frame shift mutation at residue 807 [2], both with the full-length DISC1 protein and the isolated C region. Given the numerous reports of using single-domain antibodies for structural investigation of challenging proteins through techniques such as X-ray crystallography and cryo-electron microscopy, V_H_H domains like the one described in this study may ultimately pave the way for experimental determination of the DISC1 structure, which has defied all efforts to date [61, 62].

While this manuscript was under revision, the NMR structure of a C-terminal segment of mouse Disc1 (residues 765–852, corresponding approximately to half of the C region) fused to an Ndel1 peptide (residues 238–284) was published [63]. The two moieties essentially form a three-helix coiled coil, with an extensive hydrophobic core established by apolar side chains at the *a* and *d* positions of the respective heptad repeats. Superposition of this complex with our *ab initio* model of human DISC1^691-836^ (S1 Fig) reveals good agreement within the ordered DISC1 region shared by both structures, with a root-mean-square distance of 2.32 Å for the 52 alpha carbon atoms of the helical hairpin (residues 773–800 and 806–829). Intriguingly, the position of the Ndel1 fragment in the solution structure largely overlaps with the N-terminal helix (residues 691–711) of our DISC1 model, albeit with a reversed orientation. This apparent paradox may be rationalized by considering that the helical hairpin structure on its own exposes a significant hydrophobic surface and would thus seem unlikely to be stable in solution. In the absence of an extraneous binding partner, this surface may be shielded by a complementary amphipathic segment of the DISC1 protein (in either *cis* or *trans*), and given that the human DISC1^691-836^ monomer is stable in solution [19], the native contact is likely to be intramolecular (*cis*), in agreement with our model. We therefore speculate that the extensive hydrophobic surface involved in non-constitutive interaction with NDEL1 is only exposed on demand, possibly enabling an additional layer of regulation by, for example posttranslational modifications.

## Supporting information

S1 Fig Potential interactions of the C-terminal helical hairpin of the DISC1 protein. (DOCX)
