# Supplementary material for: Biophysical insights from a single chain camelid antibody directed against the Disrupted-in-Schizophrenia 1 protein
Source: PLoS One. 2018 Jan 11;13(1):e0191162. doi: 10.1371/journal.pone.0191162 (PMC5764400; doi:10.1371/journal.pone.0191162)
Supplement: S1 Fig — (DOCX) [file pone.0191162.s001.docx]

**Supporting information**

A.S.K. Yerabham *et al.*,

**Biophysical insights from a single chain camelid antibody directed against the disrupted in schizophrenia 1 protein**


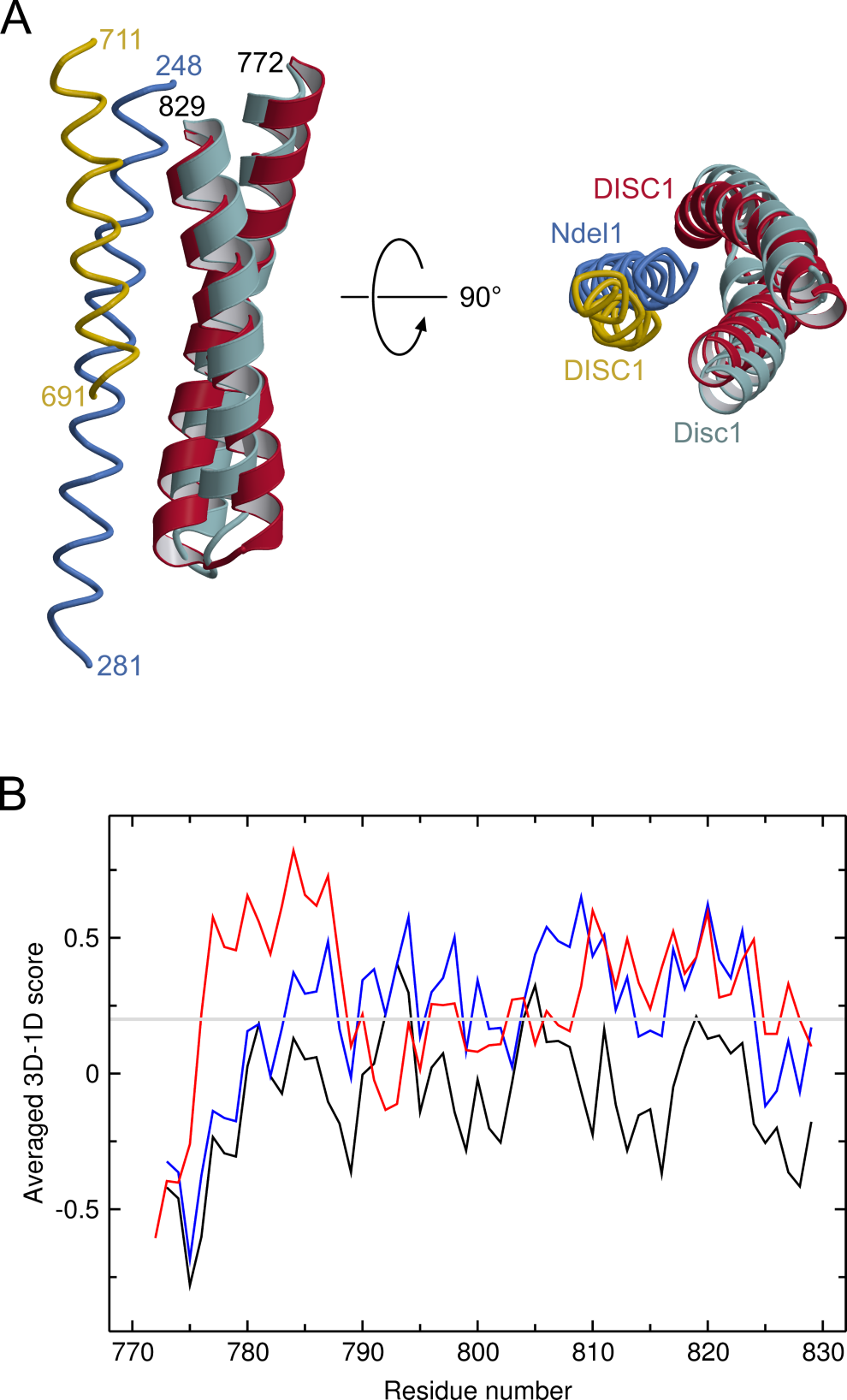


**S1 Fig. Potential interactions of the C-terminal helical hairpin of the DISC1 protein.**
(A) Residues 772-829 of our human DISC1 model (red ribbon) are superimposed on the respective segment of the mouse Disc1 structure determined by NMR spectroscopy (grey ribbon, PDB ID 5YI4). In the solution structure the hydrophobic face of the hairpin is shielded by an amphipathic helix of Ndel1 (blue trace), resulting in a three-helix coiled coil, while the model predicts an α-helix located at the N-terminus of the human DISC1 C region (res. 691-711, yellow trace) to occupy a similar position. (B) The 3D-1D score determined by *Verify3d* is a useful metric to assess the environment of individual residues in folded proteins. While the scores for the isolated helical hairpin of the mouse Disc1 structure (black) are low (indicating a non-favorable environment), they are improved significantly in the presence of the Ndel1 helix (blue). A similar stabilization is achieved in the context of the full human C region model (red) including the 691-711 helix. Scores represent averages over a five-residue sliding window centered on the residue of interest; the grey line indicates an empirical threshold (0.2) that is often used to gauge the quality of protein models.
